# Daily-life stress reactivity and recovery following virtual-reality-based cognitive behavioral therapy in patients with a psychotic disorder

**DOI:** 10.3389/fpsyt.2024.1360165

**Published:** 2024-04-30

**Authors:** Elisabeth C. D. van der Stouwe, Sanne H. Booij, Chris N. W. Geraets, Roos M. C. A. Pot-Kolder, Anna Kuranova, Mark van der Gaag, Wim Veling

**Affiliations:** ^1^ University of Groningen, University Medical Center Groningen, Department of Psychiatry, Groningen, Netherlands; ^2^ University Medical Center Groningen, University Center Psychiatry (UCP) Interdisciplinary Center Psychopathology and Emotion Regulation (ICPE), University of Groningen, Groningen, Netherlands; ^3^ Center for Integrative Psychiatry, Lentis, Groningen, Netherlands; ^4^ University of Melbourne, Centre for Youth Mental Health, Melbourne, VIC, Australia; ^5^ Department of Psychology, Health and Technology, Orygen, Melbourne, VIC, Australia; ^6^ VU University and Amsterdam Public Mental Health Research Institute, Department of Clinical Psychology, Amsterdam, Netherlands; ^7^ Parnassia Psychiatric Institute, The Hague, Netherlands

**Keywords:** VR, stress reactivity, stress recovery, paranoia, psychosis

## Abstract

**Introduction:**

Studies have consistently demonstrated increased stress sensitivity in individuals with psychosis. Since stress sensitivity may play a role in the onset and maintenance of psychosis, this could potentially be a promising target for treatment. The current study was the first to investigate whether reactivity to and recovery from daily-life stressors in psychosis change in response to treatment, namely virtual-reality-based cognitive behavioral therapy (VR-CBT).

**Methods:**

116 patients were randomized to either VR-CBT or the waiting list control group (WL). Pre-treatment and post-treatment participants completed a diary ten times a day during six to ten days. Multilevel analyses were used to model the time-lagged effects of daily stressful events on negative affect (NA) and paranoia symptoms to examine reactivity and recovery.

**Results:**

There was a significant difference in NA reactivity. VR-CBT showed higher NA at post-treatment compared to pre-treatment than WL (b_pre_=0.14; b_post_=0.19 vs b_pre_=0.18; b_post_=0.14). There was a significant difference in NA recovery and paranoia recovery between the groups at lag 1: VR-CBT showed relatively lower negative affect (b_pre_=0.07; b_post_=-0.06) and paranoia (b_pre_= 0.08; b_post_=-0.10) at post-treatment compared to pre-treatment than WL (b_pre_=0.08; b_post_=0.08; b_pre_=0.04; b_post_=0.03).

**Conclusion:**

Negative affect and paranoia recovery improved in response to treatment. Increased NA reactivity may be explained by a decrease in safety behavior in the VR-CBT group. The discrepancy between reactivity and recovery findings may be explained by the inhibitory learning theory that suggests that an original threat reaction may not erase but can be inhibited as a consequence of exposure therapy.

## Introduction

Stress sensitivity (i.e., reactivity and recovery) is a psychological mechanism that refers to the extent to which individuals react in response to stressful events. Several experience sampling method (ESM) studies have demonstrated that individuals with psychosis show increased sensitivity to daily stressful events compared to individuals without psychosis (1). More specifically, daily stressful events often precede an increase in negative affect and the occurrence of psychotic experiences (2, 3). Furthermore, Vaessen et al. (4) found that people with a psychotic disorder show a prolonged increase in negative affect and feelings of suspiciousness during recovery from stressful events compared to people from the general population. Reactivity has been investigated in people at varying points along the continuum of psychotic experiences as well. In individuals at ultra-high risk for psychosis, stress reactivity was increased (5). In the general population, increased stress reactivity predicted the persistence of psychotic experiences over the next year (6, 7). Myin-Germeys et al. (8) suggested that the level of vulnerability to psychosis mirrored the level of emotional stress reactivity. Taken together, emotional stress sensitivity (i.e., reactivity and recovery) has been shown to be an important mechanism in the pathway to psychosis (9, 10).

Emotional stress sensitivity has also been repeatedly suggested to be an important modifiable target for treatment. However, research is scarce (9). In a group of patients with chronic pain, mindfulness has shown to reduce day to day stress recovery (11). Moreover, mindfulness-based interventions have been shown to reduce emotional stress reactivity in individuals with partially-remitted depression (12). Next to mindfulness, cognitive behavioral therapy (CBT) is another likely candidate to improve emotional stress sensitivity. Specifically Virtual Reality CBT since, in VR, real-world daily life stressful situations can be simulated, and patients can receive real-time therapy and coaching to drop safety behavior during exposure to these situations. Not much is known about the potential of VR-CBT to improve either stress reactivity or recovery. However, based on the inhibitory learning theory (13), prolonged exposure may especially have a positive effect on stress recovery.

Recently, our group has developed a virtual-reality-based cognitive behavioral therapy (VR-CBT) for paranoia (14). Throughout the therapy, patients are exposed to simulations of stressful real-world daily life situations. Patients drop safety behavior, explore and challenge suspicious thoughts, and test harm expectancies to learn they are safe and can endure stressful situations. VR-CBT has already been shown to reduce average paranoid symptoms and lower levels of negative affect in daily life (15, 16). The aim of the current study was to investigate whether negative affect and paranoia reactivity and recovery are amendable by VR-CBT in individuals with psychosis. The results of such investigation may yield insight in working mechanisms of existing treatments and provide directions for development of novel interventions. To our knowledge, the current ESM study was the first to examine reactivity and recovery to stressful events in daily life in a randomized controlled design, by assessing changes following treatment. Based on previous findings, we expected reduced paranoia and negative affect reactivity and quicker paranoia and negative affect recovery after unpleasant events following VR-CBT compared to waiting list.

## Method

### Study design

The current study was a single-blind multi-center randomized controlled trial (ISRCTN registration: 12929657). Assessments were carried out pre-treatment, post-treatment (three months after pre-treatment), and at follow-up (six months after pre-treatment) by trained assessors who were blind to randomization status. In the current study, data of the pre-treatment and post-treatment assessments were used. Following pre-treatment assessment, an independent researcher who was not involved in the trial randomized participants to either VR-CBT or treatment as usual. Written informed consent was obtained from all participants. The study was approved by the ethical committee of the Amsterdam University Medical Center (METC number NL37356.058.12) and was performed in line with the declaration of Helsinki. A detailed description of the study protocol was provided by Pot-Kolder et al. (14).

### Participants

Participants were outpatients recruited from seven mental health centers in the Netherlands. The primary selection of participants was based on avoidance behavior, since VRET-P focused on exposure to situations which are previously avoided by a patient. Avoidance of particularly public transportation, bars, shops or streets was an inclusion criterion since these specific environments were available in VR. Furthermore, patients were selected based on paranoid ideations defined as a Green Paranoid Thought Scale score > 40. A moderate GPTS score was used as a cut off since in case of severe avoidance, self-reported paranoia tends to stay artificially low. Lastly, patients had to have a DSM-IV diagnosis of a psychotic disorder based on the Mini-International Neuropsychiatric Interview (17), the Schedules for Clinical Assessment in Neuropsychiatry (18), or the Comprehensive Assessment of Symptoms and History (19) (varied by center); and had to have an age of 18-65 years old. Exclusion criteria were: a history of epilepsy; IQ <70; and insufficient command of the Dutch language.

### Intervention

VR-CBT: VR-CBT for paranoia was based on parts of existing CBT protocols. However, the VR component enables therapists to gradually expose patients to controlled social environments and tailor exposure exercises to the individual patient. Thus, instead of *in vivo*, exposure exercises or behavioral experiments were performed in VR during sessions. VR-CBT consisted of 16 sessions of 60 minutes given by a trained psychologist twice a week. In the first two sessions, personalized treatment goals were set using an individualized case formulation, and participants got familiar with VR. In the subsequent sessions, 40 minutes were retained to practice with exposure, behavioral experiments, reducing safety behavior, and attention strategies in VR and 20 minutes were reserved for planning and evaluating exercises. More details of the intervention have been published previously (14).

Waiting list control group (WL): Participants in both study groups received treatment as usual. This could entail antipsychotic medication, regular contact with a psychiatrist to control symptoms, and regular contact with a psychiatric nurse to improve self-care, daytime activities, and social and community functioning.

### Experience sampling

Participants completed questionnaires for six to ten days using an electronic diary, the PsyMate^®^ (PsyMate BV, Maastricht, The Netherlands; 20). The PsyMate was programmed to generate beeps ten times a day at semi-random intervals of ±90 minutes between 7.30 AM and 10.30 PM. After every alarm beep, participants had to fill out an electronic diary. They were instructed to do so immediately after the beep, but a delay of maximum 15 minutes was allowed. On average, participants completed 47.4 (*SD* = 12.0) out of approximately 70 diary assessments. Pre-treatment this was on average 49.9 (SD = 14.0) and at post-treatment the average was 45.4 (SD = 9.7) assessments.

### Measures

Daily life stress: Participants rated items regarding the most important event that happened since the previous assessment point at each beep. The item “How pleasant was this event?” was scored on a 7-point scale ranging from -3 (“very unpleasant”) to +3 (“very pleasant”). Responses were recoded to allow high scores to reflect high perceived stress. For the current study, we only used events appraised as unpleasant (1, 2, 3) or neutral (0; reference category). Hence, the variation in intensity of appraised stress is taken into account in our approach. To assess stressful events, (level of) unpleasantness has been used often, and research has shown unpleasantness to be the strongest indicator of stressfulness, compared to other aspects of stress (e.g., predictability, controllability) (21). On average, 7.2 (SD= 8.9) unpleasant events were reported, 14.6 (SD= 13.1) neutral events were reported, and 22.9 (SD= 16.2) pleasant events were reported.

Negative affect: Negative affect (NA) was assessed by calculating the mean score of the following items: “I feel down,” “I feel anxious,” “I feel insecure,” “I feel disappointed,” “I feel lonely,” “I feel guilty,” “I feel safe” (reverse score, further referred to as “unsafe”), “I feel annoyed.” These items were rated on a 7-point Likert scale, ranging from 1 (“not at all”) to 7 (“very”). The internal consistency was calculated person by person (8 items; *α*= .96).

Paranoia: The mean score of the following paranoia items was used: **“**I feel suspicious,” “I feel that others might hurt me,” and “I feel that others dislike me,” rated on a 7-point Likert scale, ranging from 1 (“not at all”) to 7 (“very”). Internal consistency was calculated person by person (3 items; *α*= 0.95).

### Statistical analyses

By design, ESM data have a hierarchical structure; in this study, multiple observations (level 1) were nested within individuals (level 2). Therefore, we used linear mixed models that are multilevel models, including both fixed and random effects. NA, PA, paranoia, and unpleasantness, as well as assessment wave (pre and post), were time-varying variables, whereas person characteristics and group (intervention/control) were time-invariant. Time-varying predictor variables were person-mean centered to reflect within-individual deviations from the person mean (22, 23). As a sensitivity check, we also performed analyses with centering performed at the waves within the person level. This was done to check whether the results hold when any changes in mean levels over time are filtered out. In addition, because increases or decreases in measurements over time may induce spurious associations, time-varying predictors and outcomes were detrended as well, within waves (24).

Negative affect and symptom reactivity and recovery were assessed by modeling the effect of (the lagged levels of) unpleasant events on the lagged levels of negative affect and paranoia. These models were constructed for the previous five time points (25), and lags were created within days, such that there was no ‘overnight’ prediction of unpleasant events on negative affect and paranoia. *Reactivity* The first model (lag 0) was constructed to assess reactivity and included the level of negative affect or paranoia at a time point (t) as an outcome variable and the level of the unpleasantness of the event at the same time point (t) as a predictor variable.

Recovery The subsequent models (lag 1, 2, 3 and 4) were constructed to assess recovery. For example, the second model assessed the lag-1 effect with negative affect or paranoia at the time (t) as the outcome and the lagged unpleasantness of the event at the previous time point (t − 1) as the predictor, approximately 90 min earlier. Similar models were constructed for the other three time points (t – 2, t – 3, t − 4).

To assess differences in NA recovery and symptom recovery between the VR-CBT group and the WL group across pre-treatment assessment and post-treatment assessment we included a three-way interaction: group*wave*unpleasantness. Pre-intervention wave and the control group were the reference categories.

Subsequently, to assess group-specific trajectories of negative affect recovery and paranoia recovery, we assessed the effect for each group separately by removing the three-way interaction and using the two-way interaction wave*unpleasantness to assess group-specific trajectories. Models were fitted separately for the VR-CBT and WL groups for five consecutive time points.

Random intercepts and slopes were added to the model when this improved model fit. This was the case for a random intercept at the person level, which means there were individual differences in mean levels of NA or paranoia for the participants. In addition, a random slope for the time-varying predictor variables event unpleasantness and wave (dummy-coded) were included, indicating the effect of unpleasantness on NA and paranoia, as well as the effect of wave on these outcomes, differed across participants. Models with different covariance structures for the random effects and the error-covariance matrix were fitted using restricted maximum likelihood estimation, and the most optimal model was chosen based on the Akaike Information Criterion (AIC). The final models were estimated with maximum likelihood estimation. Models were calculated using STATA 14.2. In all analyses, a p-value <0.05 was considered statistically significant.

## Results

### Sample characteristics

In total, 116 participants were included in the study. Baseline characteristics of the participants from the VR-CBT group and the WL group are presented in **Table 1**. There were no significant differences between the groups at baseline. ESM response rates were at baseline on average 46.1 (*SD=* 13.3) out of 70 ESM assessments and at post-treatment assessment, on average, 43.1 (SD= 10.1).

**Table 1:** Demographical and clinical characteristics of the VR-CBT group and the WL group.

	VR-CBT N=58	WL N=58
Age	36.5 (10)	39.5 (10)
Sex (female)	18 (31%)	16 (28%)
Education		
None or primary	16 (28%)	16 (28%)
Vocational	18 (31%)	24 (41%)
Secondary	9 (16%)	9 (16%)
Higher	15 (26%)	9 (16%)
DSM-IV diagnosis		
Schizophrenia	46 (79%)	49 (85%)
Schizoaffective disorder	1 (2%)	5 (9%)
Delusional disorder	1 (2%)	0 (0%)
Psychotic disorder NOS.	10 (17%)	4(7%)
Duration of illness	13.3 (10.6)	14.9 (9.5)
Medication		
Antipsychotics	54 (93%)	57 (98%)
Olanzapine equivalent of prescribed antipsychotic medication (mg/day)	10.5 (6.8)	11.0 (8.3)
Antidepressants	15 (26%)	17 (29%)

Data are n (%) or mean (standard deviation). VR-CBT, virtual reality cognitive behavioural.

therapy; WL, waiting list control group.

### Mean levels of negative affect and paranoia

The mean levels of negative affect and paranoia at baseline and post-assessment for each group are depicted in **Table 2**. In line with findings on specific mental states in our previous paper (16), a significant treatment effect was found at post-assessment for the compiled variable paranoia: the level of paranoia significantly decreased in the VR-CBT group, and not in the WL group.

**Table 2:** Weighted means and standard deviations of the ESM variables.

	VR-CBT		WL		Time*treatment	
	Pre	Post	Pre	Post	Pre-Post	
	N= 58	N= 47	N= 57	N= 49		
	M (SD)	M (SD)	M (SD)	M (SD)	b (95% BI)	p
Negative affect	2.96 (1.01)	2.74 (1.10)	3.20 (1.29)	3.13 (1.32)	-0.27 (-0.73-0.19)	0.26
Paranoia	3.06 (1.40)	2.71 (1.38)	3.14 (1.43)	3.30 (1.60)	-0.81 (-1.36- -0.25)	**<0.01**
Event Unpleasantness	3.31 (0.82)	3.38 (0.91)	3.41 (0.85)	3.41 (0.92)	0.07 (-0.11- 0.25)	0.47

M, mean; SD, standard deviation; b, parameter estimate (regression coefficient); CI confidence interval; VR-CBT, virtual reality cognitive behavioural therapy; WL, waiting list control group; pre, baseline; post, post-treatment.

Bold values refer to significant findings.

### Negative affect and paranoia reactivity

Negative affect and paranoia reactivity were measured with the model assessing the level of affect or paranoia and unpleasantness at the concurrent time point (lag 0). Results are presented in **Table 3**. For NA, the three-way interaction between group, post-treatment assessment (wave 2), and unpleasantness was significant (p=0.02). The VR-CBT (b_pre_=0.14; b_post_=0.19) group showed an increase in NA reactivity at post-treatment assessment compared to pre-treatment assessment, while the WL group showed a decrease (b_pre_=0.18; b_post_=0.14) (see **Table 4**). The three-way interaction was insignificant for paranoia (p=0.77).

**Table 3:** Differences between groups in negative affect and paranoia reactivity and recovery.

	Estimates (95% CI)	p
NA		
Lag 0		
Unpleasantness*group*post-treatment	0.13 (0.02-0.23)	**0.02**
Lag 1		
Unpleasantness*group*post-treatment	-0.18 (-0.33 – 0.03)	**0.02**
Lag 2		
Unpleasantness*group*post-treatment	0.02 (-0.16 – 0.19)	0.85
Lag3		
Unpleasantness*group*post-treatment	-0.11 (-0.31 – 0.09)	0.28
Lag 4		
Unpleasantness*group*post-treatment	0.02 (-0.22 – 0.26)	0.86
Paranoia		
Lag 0		
Unpleasantness*group*post-treatment	-0.01 (-0.12 – 0.09)	0.77
Lag 1		
Unpleasantness*group*post-treatment	-0.25 (-0.40 – -0.09)	**<0.01**
Lag 2		
Unpleasantness*group*post-treatment	0.11 (-0.07 – 0.29)	0.23
Lag3		
Unpleasantness*group*post-treatment	-0.11 (-0.31 - 0.08)	0.26
Lag 4		
Unpleasantness*group*post-treatment	0.02 (-0.20 – 0.24)	0.88

Bold values refer to significant findings.

**Table 4:** Reactivity and recovery models separated for the VR-CBT and WL groups.

	VR-CBT					WL				
	Pre		Post		P-value two-way interaction	Pre		Post		P-value two-way interaction
	Estimate	SE	Estimate	SE	p	Estimate	SE	Estimate	SE	p
NA										
Lag 0	0.14**	0.03	0.19**	0.03	<0.01**	0.18**	0.02	0.14**	0.03	0.18
Lag 1	0.07*	0.04	-0.06	0.05	<0.01**	0.08*	0.04	0.08*	0.04	0.97
Lag 2	0.03	0.04	0.07	0.05	0.54	-0.07	0.05	0.00	0.05	0.90
Lag 3	0.03	0.05	-0.14*	0.06	0.01*	0.05	0.05	0.07	0.06	0.39
Lag 4	0.03	0.05	0.14*	0.06	0.04*	0.16**	0.05	0.22**	0.06	0.46
Paranoia										
Lag 0	0.13**	0.02	0.12**	0.03	0.45	0.17**	0.03	0.13**	0.03	0.88
Lag 1	0.08*	0.03	-0.10*	0.04	<0.01**	0.04	0.04	0.03	0.04	0.63
Lag 2	-0.05	0.04	0.05	0.04	0.08	-0.05	0.05	-0.02	0.05	0.72
Lag 3	0.07	0.05	0.12*	0.05	<0.01**	0.03	0.05	0.04	0.05	0.68
Lag 4	0.00	0.05	0.11*	0.05	0.12	-0.01	0.06	0.08	0.06	0.14

VR-CBT, virtual reality cognitive behavioural therapy; WL, waiting list control group; NA, negative affect, PA, positive affect; b, b coefficients of multilevel models.

*p-value < 0.05.

**p-value < 0.01.

Bold values refer to significant findings.

### Negative affect and paranoia recovery

Negative affect: In the lag 1 model, the three-way interaction between group, post-treatment assessment (wave 2), and unpleasantness was significant (p=0.02). VR-CBT showed a decreased negative affect level after unpleasant events at post-treatment assessment compared to pre-treatment assessment (b_pre_=0.07; b_post_=-0.06), while WL showed no change (b_pre_=0.08; b_post_=0.08). The three-way interactions between group, post-treatment assessment (wave 2) and unpleasantness were non-significant in the models for lag 2, lag 3, and lag 4 (see **Table 3**).

Paranoia: In the model of lag 1, the three-way interaction between group, post-treatment assessment (wave 2), and unpleasantness was significant (p<0.01). VR-CBT showed a decreased paranoia level after unpleasant events at post-treatment assessment compared to pre-treatment assessment (b_pre_=0.08; b_post_=-0.10), while WL stayed relatively stable (b_pre_=0.04; b_post_=0.03). In the models of lag 2, 3 and 4, the three-way interactions were non-significant.

### Group-specific trajectories of affect recovery and paranoia recovery

**Figure 1:**
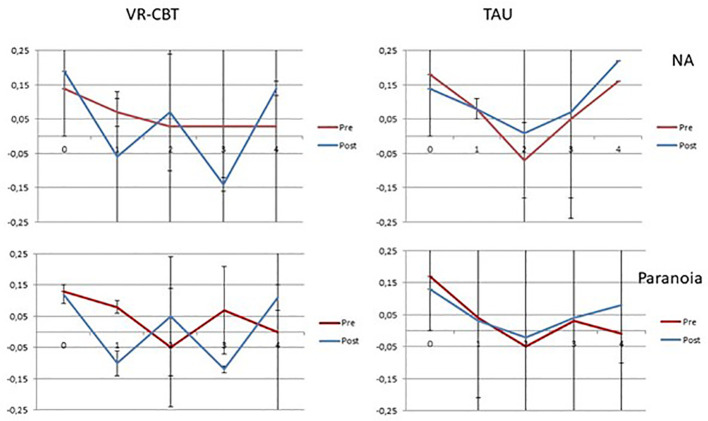
Group-specific trajectories of negative affect and paranoia reactivity and recovery for the VR-CBT and WL groups. The y-axis depicts the b-coefficients that represent the effect on negative affect and paranoia at the corresponding lags depicted on the x-axis. NA, negative affect.

Results of the analyses for the VR-CBT group and the WL group separately are presented in **Table 4**. Group-specific trajectories are depicted in **Figure 1**.

### Sensitivity analyses

In preparation for the analyses, variables were person-mean centered to reflect within-individual deviations from the person mean. In sensitivity analyses, we used variables that were wave-mean centered as well. In other words, participants’ data were adjusted to the mean of their data for each wave separately. Results of negative affect and paranoia reactivity and paranoia recovery did not differ from those of the main analyses (see **Supplementary Table 1**). Results of negative affect recovery changed in one respect. The three-way interaction between group, post-treatment assessment (wave 2), and unpleasantness was significant for lag 3 (p=0.02, as opposed to 0.28 in main analysis) (See **Supplementary Table 2** and **Supplementary Figure 1** for the group-specific estimates). At lag 3, VR-CBT showed a decrease in NA after unpleasant events at post-treatment assessment compared to pre-treatment assessment (b_pre_=0.03; b_post_=-0.14), while WL showed no change (b_pre_=0.05; b_post_=0.05).

## Discussion

We investigated changes in reactivity and recovery from stressful events in daily life in psychosis following VR-CBT compared to a waiting list control group. To our knowledge, this is the first ESM study to examine whether stress reactivity and recovery change in response to treatment. Taken together, results showed changes in stress recovery following VR-CBT in the expected direction (i.e. quicker stress recovery). With regard to reactivity, negative affect reactivity was increased following the VR-CBT group and no differences were found for paranoia reactivity.

Patients who received VR-CBT showed a 90 minutes quicker negative affect recovery and paranoia recovery after stressful events in daily life than patients in the WL group. While previous studies demonstrated that a prolonged increase in negative affect and paranoia following stressful events may play a role in the onset and maintenance of psychosis (4), the current study indicates that particularly stress recovery may indeed be amenable by VR-CBT. Looking more closely at the recovery findings, it becomes apparent that differences in recovery between both groups were significant for the first time lag and in the sensitivity analyses for the third lag as well, but not for the second and fourth lags. With regard to the fourth lag, this might be explained by the finding that pre-treatment stress recovery was already close to (non-significantly different from) baseline at 4 lags. There may have been little room to improve at this point in the recovery phase.

Whereas recovery results were in the expected direction, negative affect reactivity was increased following the VR-CBT group and no differences were found for paranoia reactivity. An explanation for increased negative affect reactivity may be that throughout VR-CBT patients are encouraged to drop safety behavior which enables exposure to high levels of fear. Indeed, compared to the control group, use of safety behaviors decreased significantly in the VR-CBT group (15). Furthermore, the discrepancy between increased reactivity and quicker recovery may be explained by the inhibitory learning theory which posits that the original CS-US association learned during fear conditioning is not erased during extinction in therapy, but rather is left intact while new, secondary inhibitory learning about the CS-US develops - specifically, that the CS no longer predicts the US (13, 26, 27). In other words, there still may be an original initial stress reaction, but this is inhibited by new learned information. The initial reactivity to stress might be a more stable trait (1) associated to trauma (10), psychosis liability and negative self-esteem (28). If this is the case, the ability to recover more quickly from a stressful situation may be a more valuable treatment target. The findings further underline the emerging view in the ESM literature that the prolonged recovery following a stressful event provides a more complete image of the stress response than initial stress reactivity (4).

Our study had several limitations. First, ESM items were rated on 7-point Likert scales, which limits the amount of variation compared to continuous ESM scales. Second, we investigated differences between groups across waves for each lag separately. In this way, we could not account for slopes and values of surrounding lags. Third, throughout the analyses we did not account for subsequent or previous stressful events. Other approaches, such as used in Vaeassen et al., would accommodate taking into account surrounding stressors. However, our approach has also some advantages over other used approaches, such as the use of the neutral category as a reference (instead of both neutral and positive events), and leaving the intensity rating of the event intact. An important strength of the current study is the implementation of ESM data in an RCT design.

In conclusion, while a body of previous research has indicated that stress reactivity and recovery may play a role in the onset and maintenance of psychosis, this study adds to this knowledge by providing presumptive evidence that paranoia and negative affect recovery improve in response to treatment, while initial reactivity may be harder to change. These findings are in line with the inhibitory learning theory that suggests that an original threat reaction may not erase but can be inhibited as a consequence of exposure therapy. Our findings cautiously imply that stress sensitivity may be a clinically relevant treatment target. The current study also showed the relevance and possibility of investigating stress reactivity and recovery as an outcome in a randomized controlled trial. Future studies should replicate and investigate stress sensitivity following other interventions targeted at stress and affect to allow for more definite conclusions.

## Data availability statement

The raw data supporting the conclusions of this article will be made available by the authors, without undue reservation.

## Ethics statement

The studies involving humans were approved by Medisch ethische commissie (METc) Groningen. The studies were conducted in accordance with the local legislation and institutional requirements. The participants provided their written informed consent to participate in this study.

## Author contributions

EV: Conceptualization, Data curation, Formal analysis, Methodology, Visualization, Writing – original draft, Writing – review & editing. SB: Conceptualization, Formal analysis, Writing – original draft, Writing – review & editing. CG: Investigation, Methodology, Writing – review & editing. RP: Investigation, Project administration, Writing – review & editing. AK: Formal analysis, Writing – review & editing. MV: Conceptualization, Funding acquisition, Methodology, Resources, Writing – review & editing. WV: Conceptualization, Funding acquisition, Investigation, Methodology, Writing – original draft, Writing – review & editing.
