# Supplementary material for: Daily-life stress reactivity and recovery following virtual-reality-based cognitive behavioral therapy in patients with a psychotic disorder
Source: Front Psychiatry. 2024 Apr 30;15:1360165. doi: 10.3389/fpsyt.2024.1360165 (PMC11091723; doi:10.3389/fpsyt.2024.1360165)

**Supplementary table 1. Differences between groups in affect and paranoia reactivity and recovery.**

|  | Estimates (95% CI) |  | p |
| --- | --- | --- | --- |
| *NA* |  |  |  |
| Lag 0 |  |  |  |
| Unpleasantness*group*period2 | 0.12 (0.02-0.23) |  | **0.03** |
| Lag 1 |  |  |  |
| Unpleasantness*group*period2 | -0.22 (-0.37 - -0.07) |  | **<0.01** |
| Lag 2 |  |  |  |
| Unpleasantness*group*period2 | -0.01 (-0.18 – 0.16) |  | 0.93 |
| Lag3 |  |  |  |
| Unpleasantness*group*period2 | -0.23 (-0.42 – -0.04) |  | **0.02** |
| Lag 4 |  |  |  |
| Unpleasantness*group*period2 | -0.02 (-0.24 – 0.20) |  | 0.87 |
| *Paranoia* |  |  |  |
| Lag 0 |  |  |  |
| Unpleasantness*group*period2 | 0.02 (-0.09 – 0.12) |  | 0.77 |
| Lag 1 |  |  |  |
| Unpleasantness*group*period2 | -0.19 (-0.34 – 0.05) |  | **<0.01** |
| Lag 2 |  |  |  |
| Unpleasantness*group*period2 | 0.17 (0.00 – 0.03) |  | 0.06 |
| Lag3 |  |  |  |
| Unpleasantness*group*period2 | -0.15 (-0.33 - 0.03) |  | 0.10 |
| Lag 4 |  |  |  |
| Unpleasantness*group*period2 | 0.12 (-0.09 – 0.33) |  | 0.26 |

**Supplementary table 2**. Reactivity and recovery models separated for the VR-CBT and TAU group.

|  | **VR-CBT** |  |  |  |  | **TAU** |  |  |  |  |
| --- | --- | --- | --- | --- | --- | --- | --- | --- | --- | --- |
|  | **Pre** |  | **Post** |  | **P-value two-way interaction** | **Pre** |  | **Post** |  | **P-value two-way interaction** |
| **NA** |  |  |  |  |  |  |  |  |  |  |
| Lag 0 | **0.14**** | 0.03 | **0.20**** | 0.03 | **0.03*** | **0.17**** | 0.03 | **0.14**** | 0.03 | 0.38 |
| Lag 1 | **0.07*** | 0.04 | -0.06 | 0.05 | **0.01*** | 0.06 | 0.04 | 0.06 | 0.04 | 0.51 |
| Lag 2 | 0.03 | 0.04 | 0.07 | 0.05 | 0.28 | -0.08 | 0.05 | -0.01 | 0.05 | 0.57 |
| Lag 3 | 0.03 | 0.05 | **-0.14*** | 0.06 | **0.04*** | 0.02 | 0.05 | 0.05 | 0.06 | 0.74 |
| Lag 4 | 0.02 | 0.05 | **0.15*** | 0.06 | **0.14** | **0.14*** | 0.06 | **0.20**** | 0.07 | 0.20 |
| **Paranoia** |  |  |  |  |  |  |  |  |  |  |
| Lag 0 | **0.13**** | 0.03 | **0.13**** | 0.03 | 0.59 | **0.15**** | 0.03 | **0.13**** | 0.02 | 0.10 |
| Lag 1 | **0.08*** | 0.03 | **-0.10*** | -0.04 | **<0.01**** | 0.04 | 0.04 | 0.02 | 0.05 | 0.78 |
| Lag 2 | -0.05 | 0.04 | 0.07 | 0.04 | **0.04*** | -0.05 | 0.05 | -0.06 | 0.05 | 0.42 |
| Lag 3 | 0.06 | 0.05 | **-0.12*** | 0.05 | **0.07** | 0.02 | 0.06 | 0.03 | 0.05 | 0.54 |
| Lag 4 | -0.01 | 0.05 | **0.14*** | 0.06 | 0.20 | -0.02 | 0.06 | 0.07 | 0.06 | 0.79 |

*Note:* VR-CBT, virtual reality cognitive behavioural therapy; TAU, treatment as usual; NA, negative affect, PA, positive affect; b, b coefficients of multilevel models.

*p-value < 0.05

**p-value < 0.01

**Supplementary figure 1.** Group-specific trajectories of negative and positive affect and paranoia reactivity and recovery for the VR-CBT group and the TAU group. The y-axis depicts the b-coefficients that represent the effect on negative affect, positive affect and paranoia at the corresponding lags, which are depicted on the x-axis.


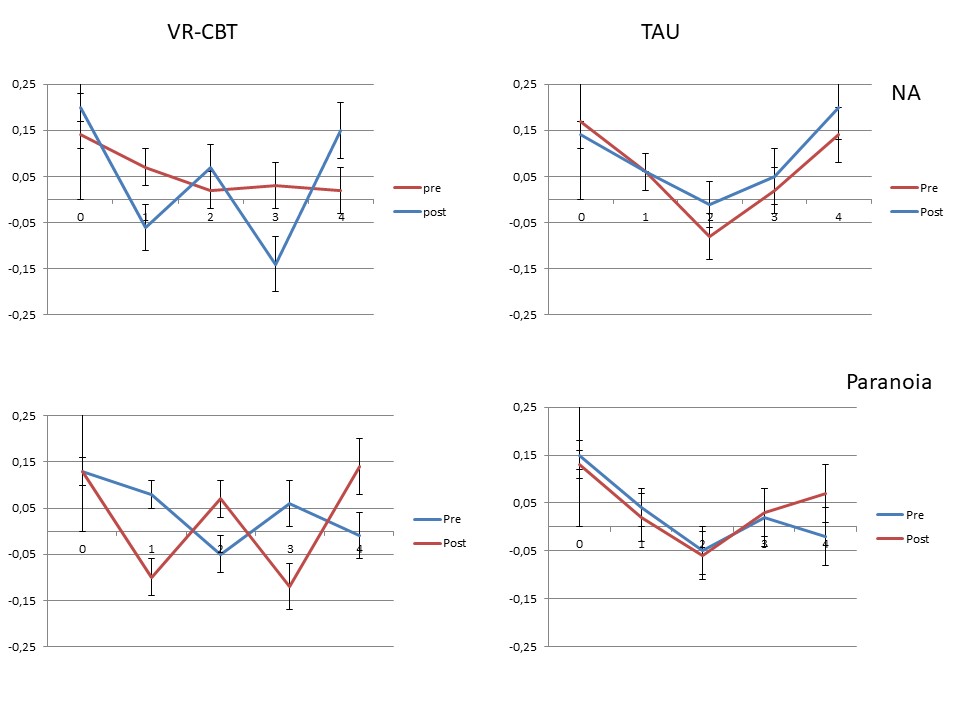

Supplement: Supplementary file 1 [file DataSheet_1.docx]
